# Prediction of the output factor using machine and deep learning approach in uniform scanning proton therapy

**DOI:** 10.1002/acm2.12899

**Published:** 2020-05-17

**Authors:** Hardev S. Grewal, Michael S. Chacko, Salahuddin Ahmad, Hosang Jin

**Affiliations:** ^1^ Oklahoma Proton Center Oklahoma City OK USA; ^2^ Department of Radiation Oncology University of Oklahoma Health Sciences Center Oklahoma City OK USA

**Keywords:** gaussian process regression, machine and deep learning, proton therapy, shallow neural network

## Abstract

**Purpose:**

The purpose of this work is to develop machine and deep learning‐based models to predict output and MU based on measured patient quality assurance (QA) data in uniform scanning proton therapy (USPT).

**Methods:**

This study involves 4,231 patient QA measurements conducted over the last 6 years. In the current approach, output and MU are predicted by an empirical model (EM) based on patient treatment plan parameters. In this study, two MATLAB‐based machine and deep learning algorithms — Gaussian process regression (GPR) and shallow neural network (SNN) — were developed. The four parameters from patient QA (range, modulation, field size, and measured output factor) were used to train these algorithms. The data were randomized with a training set containing 90% and a testing set containing remaining 10% of the data. The model performance during training was accessed using root mean square error (RMSE) and R‐squared values. The trained model was used to predict output based on the three input parameters: range, modulation, and field size. The percent difference was calculated between the predicted and measured output factors. The number of data sets required to make prediction accuracy of GPR and SNN models' invariable was also evaluated.

**Results:**

The prediction accuracy of machine and deep learning algorithms is higher than the EM. The output predictions with [GPR, SNN, and EM] within ± 2% and ± 3% difference were [97.16%, 97.64%, and 92.95%] and [99.76%, 99.29%, and 97.18%], respectively. The GPR model outperformed the SNN with a smaller number of training data sets.

**Conclusion:**

The GPR and SNN models outperformed the EM in terms of prediction accuracy. Machine and deep learning algorithms predicted the output factor and MU for USPT with higher predictive accuracy than EM. In our clinic, these models have been adopted as a secondary check of MU or output factors.

## 1 Introduction

Machine and deep learning techniques combine the power of computer science and statistics, and have the potential to revolutionize cancer treatment technologies in radiation oncology.^1^ Proton beam therapy having desirable properties such as sharp dose falloff after the Bragg peak has shown reduced dose to critical organs while achieving comparable dose to the target volume as conventional radiation therapy.^2^, ^3^ Utilizing machine and deep learning techniques in proton therapy will lead to the development of new tools that can benefit patient care.^4^, ^5^

In the uniform scanning proton therapy (USPT) delivery technique, the beam is delivered uniformly layer by layer by varying the beam energy. Scanning magnets are used to move the beam in two dimensions along the transverse plane. In radiation therapy, monitor units (MU) and the output factor (OF), which is a ratio of measured dose for a certain field with respect to a reference field, are required for the accurate and reliable delivery of the treatment dose to the patient. Due to the complexity of the USPT beam delivery, no commercial treatment planning system is available to calculate MU.

The MU and OF are calculated in both USPT and passively double‐scattered proton therapy (DSPT) from results of measurements, empirical models (EMs), or Monte Carlo simulation of each patient‐specific treatment field. There are several publications available which report the calculations of OF for DSPT and USPT techniques. For DSPT, Kooy et al.^6^, ^7^ reported the analytical method for the calculation of OF by combining the parameters of range, modulation, and source shift change. The OF calculation procedure for DSPT at M.D. Anderson Cancer Center was reported by Sahoo et al.^8^ Ferguson et al.^9^ compared the three output prediction models named, Sahoo et al.'s correction‐based model, Kooy et al.'s analytical model, and a quartic polynomial fit model for a compact double scattered proton therapy system. They concluded that the differences among these models were statistically insignificant. Zhao et al.^10^ at Midwest Proton Radiotherapy Institute (MPRI) studied the sector integration method for proton output calculations in a USPT technique. They reported that this method improved accuracy for higher energy, larger spread out Bragg peaks, and large field sizes. For USPT, Zheng et al.^11^ reported the calculation of OF for various beam conditions at the Oklahoma Proton Center. The use of the machine learning approach in intensity modulated radiation therapy (IMRT) quality assurance (QA) is published by Valdes et al.^12^, ^13^ They suggested that the machine learning based on virtual IMRT QA method provided a framework for the integration of the task group (TG) 100's risk‐based QA program. The use of the machine learning approach for the calculation of MU was reported by Sun et al.^4^ for DSPT technique.

To the best of our knowledge, there is no published work regarding the use of machine and deep learning algorithms for the calculation of OF for USPT technique. Accordingly, the purpose of this study was to detail the procedure for the use of such algorithms for the calculation and verification of OF and MU for the USPT technique. This work demonstrates the potential of machine and deep learning techniques to directly impact patient care in proton therapy.

## 2 Materials and Methods

### 2.A Patient and proton system

A total of 4,231 patient‐specific field QA measurements conducted over a span of 6 years (2013–2018) were used in this study. All measurements were performed using an IBA proton therapy system (Louvain‐la‐Neuve, Belgium). The description of the beam line has been detailed elsewhere.^11^, ^14^ The proton system can deliver beam modulation widths (proximal 95% to distal 95% isodose point) from 2.0 to 13.0 g/cm^2^ and 13.0 to 25.0 g/cm^2^ in increments of 0.5 and 1.0 g/cm^2^, respectively, and beam ranges (depth of the distal 90% isodose point) from 4.0 to 31.5 g/cm^2^ in increments of 0.1 g/cm^2^. The patient QA data include proton ranges from 4.0 to 31.5 g/cm^2^, modulation widths from 2.0 to 25.0 g/cm^2^ and field sizes from 2 × 2 to 26 × 26 cm^2^. The field size was estimated by square root of the product of the x‐axis and y‐axis at the opening of the aperture. The patient QA measurements were performed using four snout sizes: 10 cm diameter, 18 cm diameter, 25 cm diameter, and 30 × 40 cm^2^.

### 2.B Output factor measurements and empirical model

The empirical model (EM) used for the calculation of patient‐specific output factor and MU is described by Zheng et al.^11^ The EM model was developed based on various treatment conditions using combinations of ranges and modulations with the 10 cm aperture. The measured OF was then modified with several multiplicative factors such as field size, inverse square correction, and scanning field size that were empirically determined. The OF and MU from the EM for each patient‐specific field were verified by measurements using a water tank of dimensions 32 × 21×21 cm^3^ and a parallel plate ionization chamber (PPC05, IBA dosimetry, Schwarzenbruck, Germany). In our patient QA approach, the ionization chamber collected the charge (C_ref_) first for the reference condition (range: 16.0 g/cm^2^, modulation width: 10.0 g/cm^2^, MU_ref_: 150, and air‐gap: 7 cm), and then the charge (C_pt_) for each patient‐specific field using the same range, modulation, MU_pt_ as prescribed in the treatment plan and EM with field specific apertures, excluding the compensator. The measured output factor is then calculated as:

(1) $$OF_{\text{meas}}=\frac{C_{\mathrm{ref}}}{MU_{\mathrm{ref}}}÷\frac{C_{\mathrm{pt}}}{MU_{\mathrm{pt}}}$$

If the measured OF agreed with the calculation of EM within 3% for each field and had a weighted average less than 2% for all fields, the model OF and MU were used. If the measurements disagreed with the model calculation over 3% for each field or 2% overall, OF and MU were assigned based on the measurements.

### 2.C Machine and Deep learning models

**Fig. 1:**
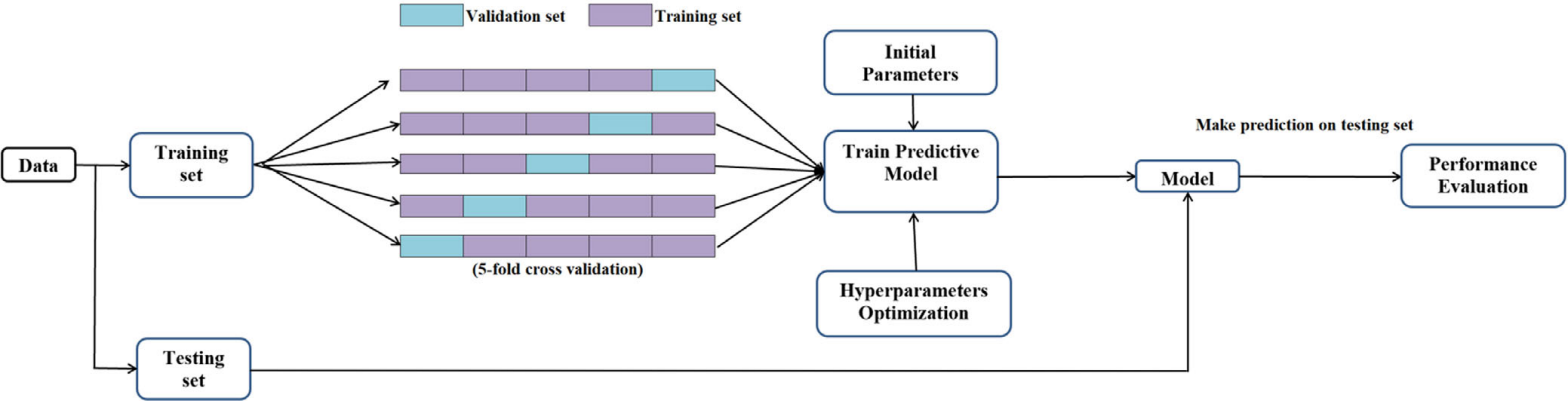
Supervised learning algorithm approach for gaussian process regression.

Since our problem involved nonlinear data fitting and multidimensional data, two MATLAB (The MathWorks, Natick, MA; version 2018b)‐based machine and deep learning algorithms — Gaussian process regression (GPR) and shallow neural network (SNN) — using supervised learning method were developed. The supervised learning model approach for GPR is shown in Fig. 1. In this study, we distinguishably used “machine learning (GPR)” and “deep learning (SNN)” as used in MATLAB even if deep learning can be viewed as an extension or a subset of machine learning techniques. The four parameters from patient QA (range, modulation, field size, and measured output factor) were used to train these algorithms. The data were randomized and the models were developed with a training set containing 90% of the data and a testing set of remaining 10%.

#### 2.C.1 Gaussian process regression

The GPR model, a form of Bayesian nonlinear regression, attempts to predict a response variable from input variables using an approach that yields certain advantages.^15^, ^16^ Given a set of training data, classical algorithms attempt to fit a single model to the data by optimizing several parameters; the model is then used to predict future inputs. Such an approach characterized by discrete parameters is known to be parametric. In contrast, GPR is nonparametric, meaning that it is not bounded by any single function, but can calculate a probability distribution over every possible function that presumably may fit the input data. Naturally, this also leads to the added benefit of obtaining uncertainty information regarding the algorithm's predictions.

The Gaussian process is defined by two functions: the mean function that outputs the expected value and the covariance function that defines how it changes as the input changes (smoothness) given that the variables included in the model come from a joint, multivariate Gaussian distribution. The mean function in this model was set to be a constant value. The covariance function can be defined as a kernel, selected to be exponential in this model, and is parameterized by a set of kernel parameters commonly known as hyperparameters. The nonisotropic exponential kernel used in the model has all predictor variables with their own correlation length scale. The exponential kernel is defined as:

(2) $$\text{k(}x_{i},x_{j})=\sigma_{f}^{2}\exp\left(\frac{-r}{\sigma_{l}}\right)$$

where *x_i_* and *x_j_* are values in input space, *σ_f_* is the signal standard deviation, *σ_l_* is the characteristics length scale and *r* is the Euclidean distance between *x_i_* and *x_j_*.

(3) $$r=\sqrt{{\left(x_{i}-x_{j}\right)}^{T}\left(x_{i}-x_{j}\right)}$$

The GPR model implemented in MATLAB can be trained via the *fitrgp* function.^17^ Observation values are projected into a feature space using basis functions. Given the basis function, the covariance function, and the initial parameter values, the *fitrgp* function estimates basis function coefficients, the noise variance, and the hyperparameters (signal variance and characteristics length scale) for the covariance kernel.

#### 2.C.2 Shallow neural network

**Fig. 2:**
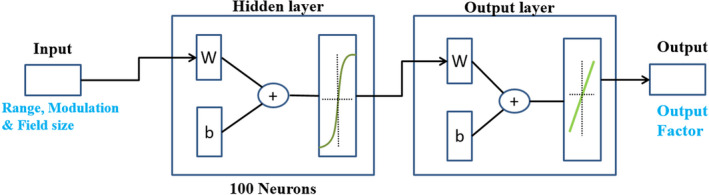
Shallow neural network design, W, weight; b, bias.

The shallow neural network as the name suggests uses fewer number of hidden layers, usually one hidden layer and one output layer as shown in Fig. 2. The number of neurons in the hidden layer may vary. Neural networks which are sets of algorithms or computing models mimicking the neural network structured in the brain can be trained for pattern recognition, classifying data, and regression problems which describe the relationship between output and one or more input variables.

A two‐layer feed‐forward SNN was constructed in this fitting problem.^18^ The hidden layer used 100 neurons with the tan‐sigmoid transfer function due to the inherent nonlinearity of our data. The sum of the weighted inputs and bias is used as the input to the tan‐sigmoid transfer function. The output layer uses the linear transfer function.

For optimal neural network training, the 90% training data set was divided into three subsets: training (80%), validation (15%), and testing (5%). The training set was used to compute the gradient and updating the network's weights and biases. During training process, the validation set error was monitored and decreased. To avoid overfitting, which is characterized by the rise in the validation set error, weights and biases were saved at the minimum of the validation error. To optimize the performance function for training multilayer feed‐forward network, a Levenberg–Marquardt back‐propagation algorithm^19^, ^20^ was used.

### 2.D Dependency of the size of the data set

In order to determine how many data points were needed to train the GPR and SNN models to make their output factor prediction accuracy invariable, the output prediction by the models was tested with 1%, 5%, 10%, 20%, 40%, 80%, and 100% of the training data set.

### 2.E Algorithm evaluation

**Fig. 3:**
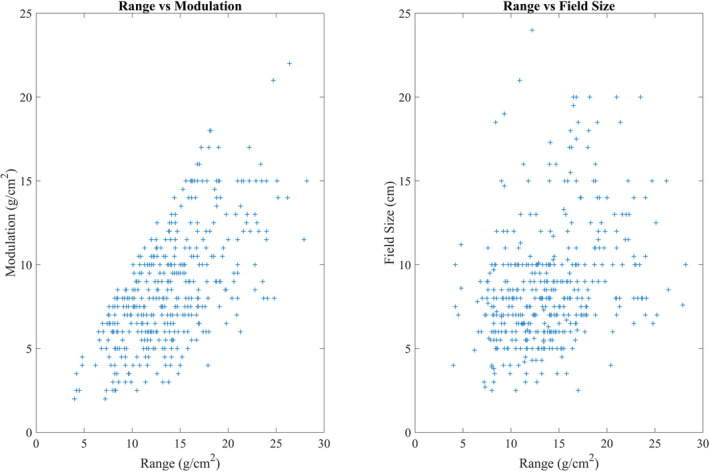
Testing data set used to make trained model predictions.

The algorithm performance during training was assessed using root mean square error (RMSE) and R‐squared values. The trained model was used to make the output predictions based on the three input parameters: range, modulation, and field size. The testing data set is shown in Fig. 3. Percent difference was calculated between the predicted and measured output factor from the testing data set. A two‐tailed t‐test was performed using a significance level of 0.05.

## 3 Results

### 3.A Output prediction and model comparison

**Fig. 4:**
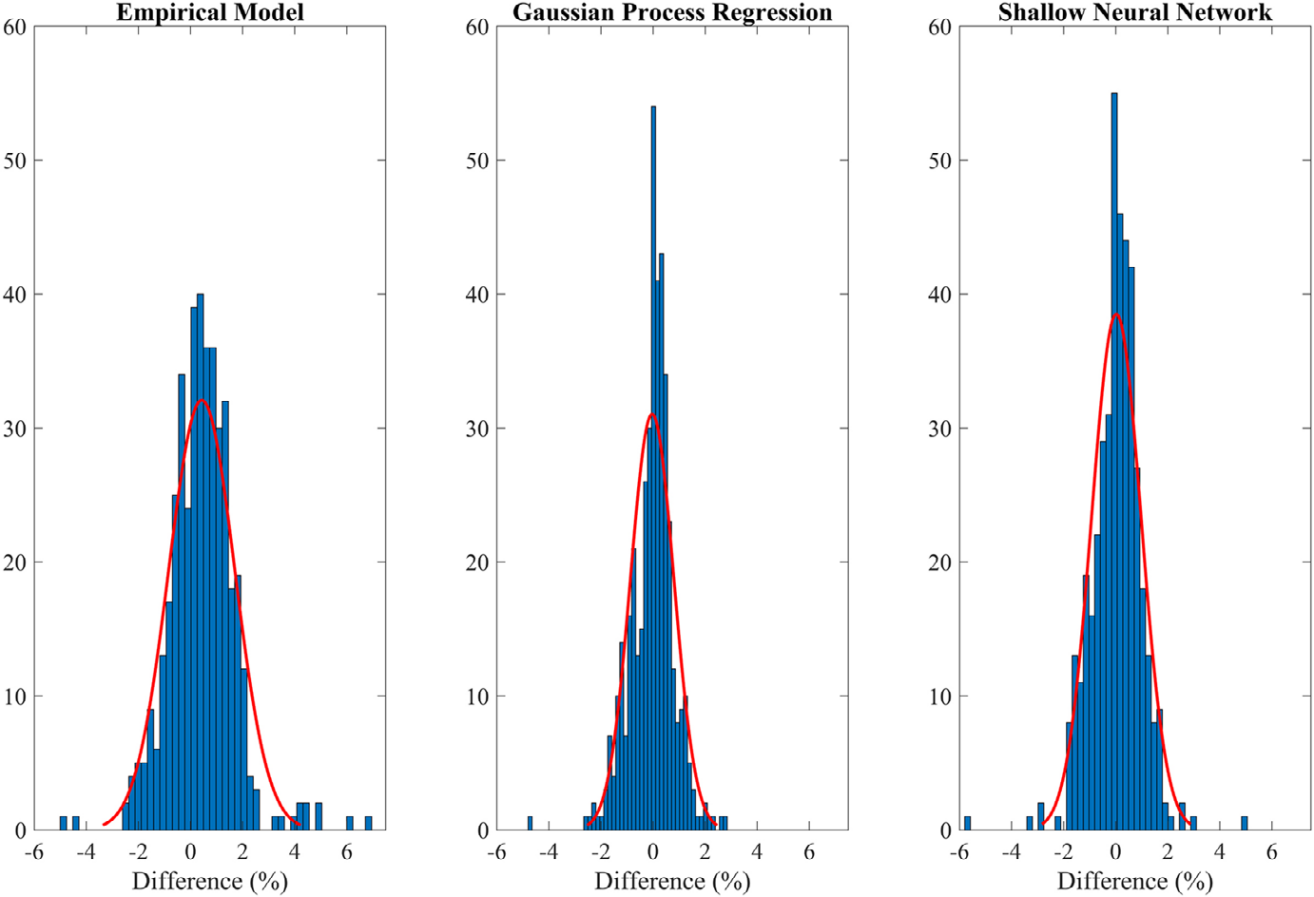
Histogram of the percent difference between measured and predicted output factors for empirical model, gaussian process regression, and shallow neural network.

Table I shows the percentage of output factor predictions that were within ± 2% and ± 3% as compared to the measured values for EM, GPR, and SNN. Table I also shows the mean absolute error with standard deviation (SD), mean error with SD, and maximum absolute error for these models. Prediction accuracy of both machine and deep learning algorithms were higher than the EM. Both GPR and SNN demonstrated prediction accuracy of greater than 97% for output factor difference within ± 2% as compared to the 92.95% for EM. All three models showed prediction accuracy of greater than 97% for difference between predicted and measured output factors to be within ± 3%. The GPR model outperformed the other two models with the least mean absolute error, SD, and maximum absolute error. The difference between GPR and SNN was not statistically significant (*P* = 0.415). However, the difference between EM and (GPR or SNN) was statistically significant (*P* < 0.001). Figure 4 shows the histogram and normal distribution fit of the percent differences between modeled and measured output factors for all patient QA data for EM, GPR, and SNN.

**Table 1:** Percent difference, mean and maximum error, standard deviation (SD) between measured and predicted output factor.

	Empirical model (%)	Gaussian process regression (%)	Shallow neural network (%)
Difference within 2%	92.95	97.16	97.64
Difference within 3%	97.18	99.76	99.29
Mean absolute error ± SD	0.92 ± 0.89	0.61 ± 0.57	0.69 ± 0.65
Mean error ± SD	0.43 ± 1.26	−0.03 ± 0.83	0.02 ± 0.95
Maximum absolute error	6.9	4.8	5.6

**Fig. 5:**
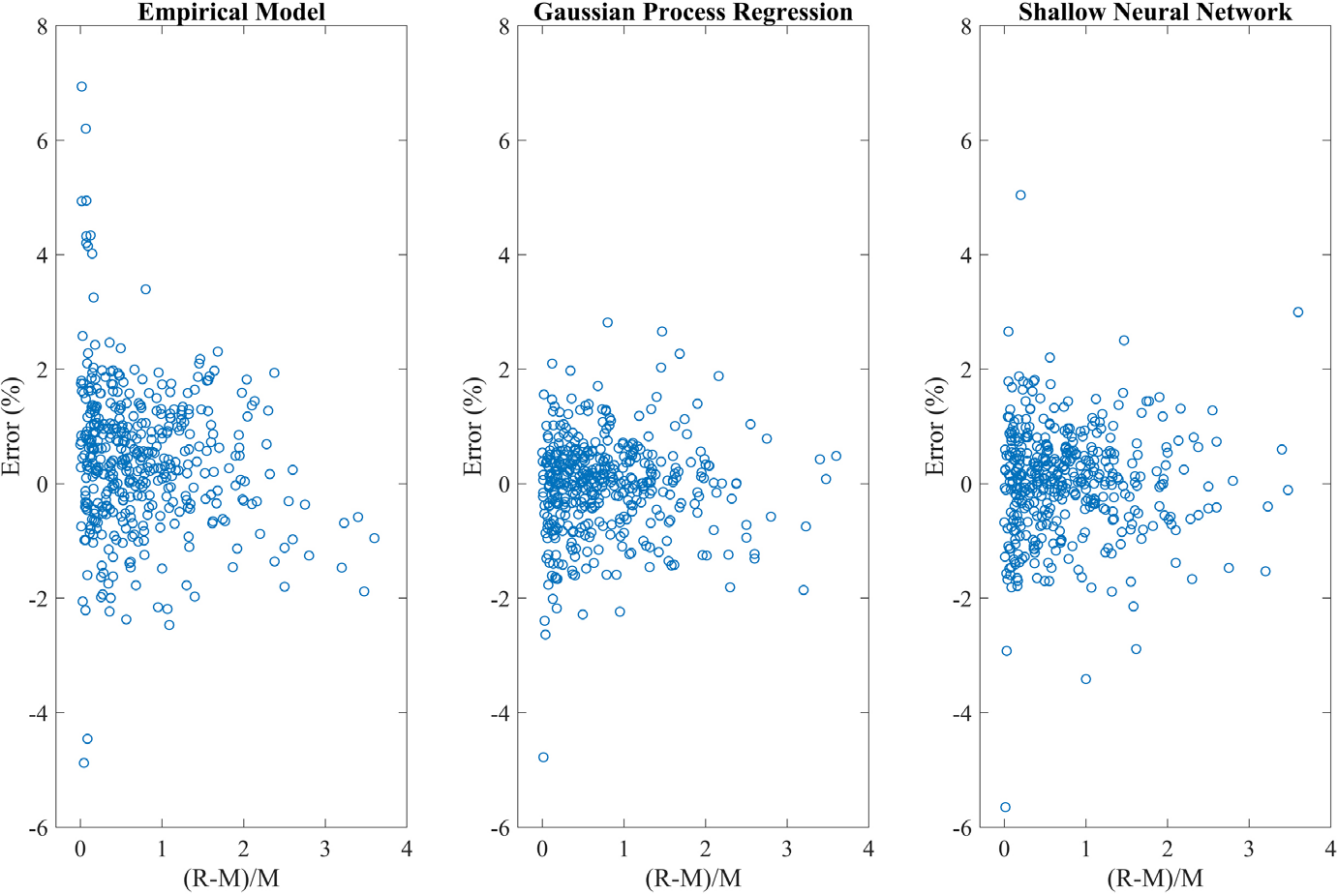
Error distribution for empirical model, gaussian process regression, and shallow neural network as a function of r = (R‐M)/M.

Figure 5 shows the distribution of the error versus *r*, where *r* = (R‐M)/M (R: proton range, M: beam modulation width) for all the three models. The small *r*‐values where range is close to the modulation show the maximum percent difference error. EM has considerably more error for small *r*‐values as compared to GPR and SNN, having a maximum absolute error of 6.9% as compared to 4.8% and 5.6% for GPR and SNN, respectively, for small *r*‐values. These findings are consistent with the results for DSPT reported by Sun et al.^4^ The percent difference errors are scattered more symmetrically around zero for GPR and SNN. The shape of the error plot for EM changes moving from left to right, which indicates the need of improvement in the model.

In the testing data set, eight data points had field size less than or equal to 3 cm. For these data points, SNN and EM had one result more than ± 2% difference between measured and predicted OF, but GPR had all results less than ± 2%. None of the model had ± 3% or more difference between measured and predicted OF for field size of 3 cm or less. For the field size greater than 15 cm (27 data points), OF predictions with [GPR, SNN, and EM] within ± 2% and ± 3% difference were [92.6%, 92.6%, and 77.8%] and [100%, 96.3%, and 88.9%], respectively.

### 3.B Dependency of the size of the data set

**Fig. 6:**
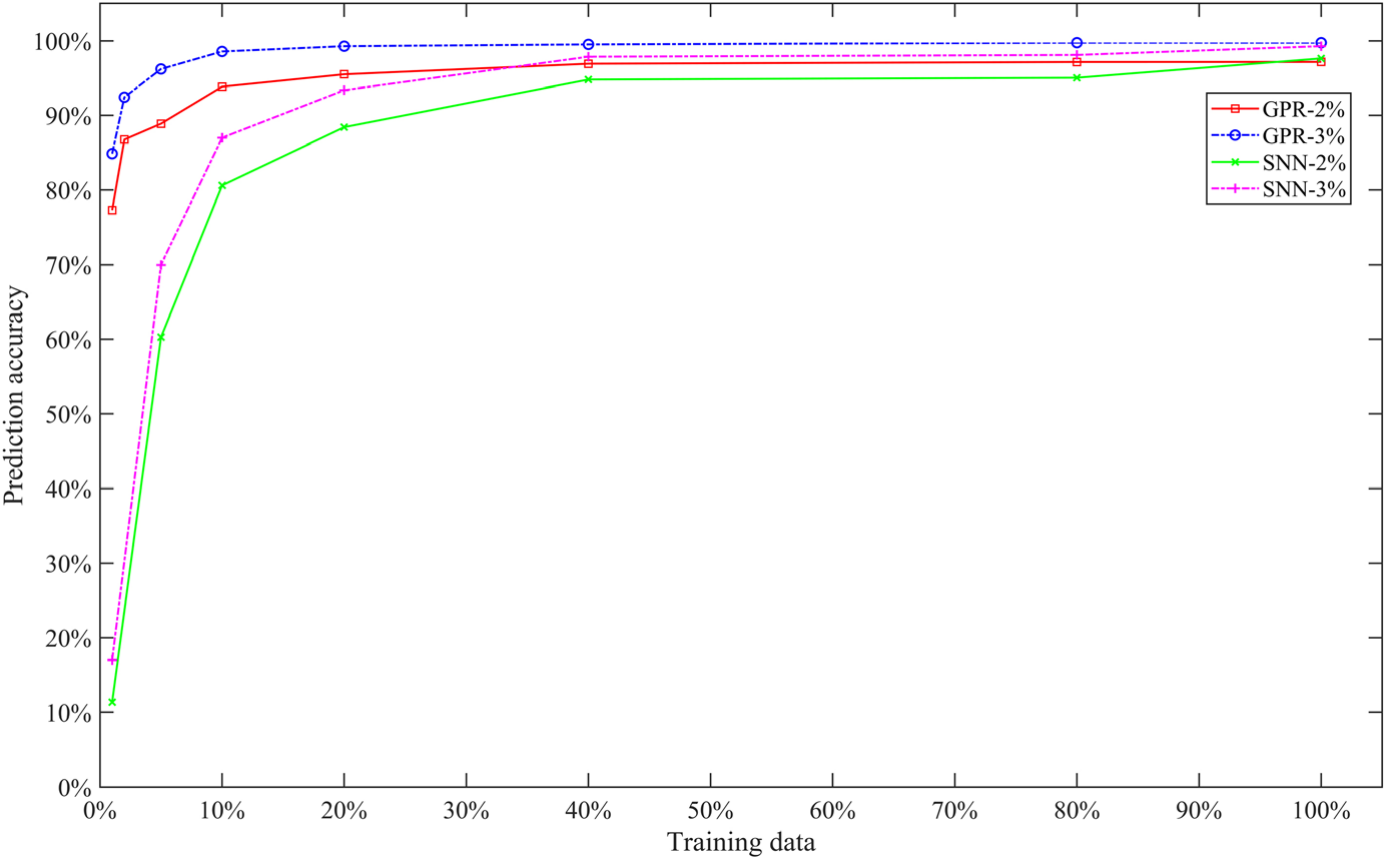
Output factor prediction accuracy as a function of training data.

The GPR model outperformed the SNN model for the sets which contained a smaller number of training data points. As the training set varied from 1% to 100% of all data points, the percentage of OF predictions within ± 2% from the measurements varied from 77% to 97% and 11% to 97% of fields for GPR and SNN, respectively. Both the models became saturated in output factor prediction accuracy at the 40% of the training data set mark as shown in Fig. 6.

## 4 Discussion

In radiation therapy, the success of the treatment is primarily dependent upon accurate dose calculation. In USPT, the conversion of the dose to MUs through the OF is presently accomplished by empirical models. Based on the patient's treatment plan QA data, two machine and deep learning models (GPR and SNN) were employed for the OF prediction. Our results indicate that both models outperformed the EM in parameters of mean error, mean absolute error, maximum absolute error, and difference within ± 2% and ± 3% of modeled OF. These encouraging results led to the development of a MATLAB compiled executable application for use in our clinic as a secondary check of MUs.

As implemented clinically, the current EM quantifies each of three features (range, modulation, and field size) into their relative contribution to the final OF. In this interpolative approach, each feature must be measured holding the other constants; as such, the incorporation of more data for treatment fields is cumbersome, requiring multiple measurement setups to quantify each individual feature alone. The machine learning approach, by contrast, allows incorporation of new data quickly, without the need for interpolation across multiple features.

Both the GPR and SNN models reported accuracy to over 97% of fields for a 2% difference between predicted and measured OF. The Cubist machine learning model developed by Sun et al.^4^ for their DSPT reported 97.5% prediction accuracy for a difference within 2% between predicted and measured OFs.

One of the limitations of the machine and deep learning approach is the need of significant amount of data points in order to build an acceptable model. Both of the models become saturated in prediction accuracy utilizing 40% of the training data set, which corresponds to approximately 1500 data points. Even though the GPR model needed only approximately 750 data points to achieve a prediction accuracy of more than 95%, Sun et al.^4^ reported that the implementation of the Cubist machine learning model required 1200 measured OFs. The results indicate that GPR needs less data points than the Cubist model, but SNN needs more to build an accurate model. The machine and deep learning models were built without curating the training data set. Patient data used for the training were not uniformly distributed across all model parameters as a result of the nonuniform distribution of disease sites treated. For example, most prostate plans were concentrated in the 24–27 g/cm^2^ range with 6–8 g/cm^2^ modulation width and ~ 7×7 cm^2^ field size. In contrast, the empirical model was built with 338 individual measurements by uniformly covering the entire range and modulation width. As a result, it is difficult to directly contrast the number of measurement points necessary between these models; however, it indicates that the empirical model requires fewer measurements to commission.

The daily output variation of our cyclotron‐based proton machine is less than 2%. This deviation may add an additional noise to the patient‐specific OF measurements. To correct this deviation, the OF of the reference field (Range: 16 cm, Modulation: 10 cm, MU: 150, Aperture size: 10 cm diameter, and Air gap: 7 cm) is measured before the start of patient‐specific QA. This reference field OF is used to normalize the patient‐specific OF.

The field size factor is interdependent upon a number of factors including range, modulation, snout position, and calibration depth. Zheng et al.^21^ reported that by varying the field size from 10 to 3 cm diameter, the field size factor decreased over 10% for high energy proton beam. For accurate dose delivery, OF must include dependence on the field size factor. One of the limitations of the Cubist machine learning model developed by Sun et al.^4^ is that the field size factor was not included in model development. Both GPR and SNN models include the field size for the model development, as defined at the isocenter plane. This plane is independent of the snout position, which was excluded from the current model development as it was by Sun et al.^4^

The EM yields large errors in predicting OF when modulation is nearly equal to the proton beam range. Similar results were reported by Kim et al.^22^ when r < 0.3, OF had large prediction uncertainty. The reason behind this mismatch is due to the uncertainty involving modulation width (proximal 95% to distal 95% dose point). When the modulation width is nearly equivalent to the beam range, the shape of the depth dose curve is nearly flat from entrance to distal 95% dose point. This led to the large uncertainty in determining the modulation width and therefore the OF. The GPR and SNN models show less sensitivity to small *r* values. Moreover, percent difference errors were symmetrically scattered about zero, indicating that both models had nearly the same probability of over‐ and underpredicting the OF.

The machine and deep learning models have also a potential for secondary output check or patient‐specific QA prediction for pencil‐beam scanning (PBS) systems. However, the main challenge is the number of input parameters for PBS systems compared to that of DS or US proton systems. OF of DS or US systems depends on only three main input parameters (range, modulation width, and field size) for each beam while OF of the PBS system depends on too many input parameters (spot position, spot energy, spot spacing, energy layer spacing, spot per MU, field optimization technique (single‐field or multiple‐field), and so on). Generalization of these input parameters is a successful key to build an acceptable OF prediction model for PBS.

## 5 Conclusion

The accurate conversion of the planned dose to the machine readable monitor units is one of the most important parts of treatment planning in uniform scanning proton therapy. We developed two output prediction methods based on machine and deep learning algorithms using GPR and SNN. Both of these algorithms outperformed the empirical model. In our clinic, these models have been used as secondary check of MU or output factor.

## Conflict of interest

There is no conflicts of interest.
